# Supplementary material for: Precise Regulation of the TAA1/TAR-YUCCA Auxin Biosynthesis Pathway in Plants
Source: Int J Mol Sci. 2023 May 10;24(10):8514. doi: 10.3390/ijms24108514 (PMC10218526; doi:10.3390/ijms24108514)
Supplement: Supplementary file 1 [file ijms-24-08514-s001.zip › ijms-2332772-supplementary.pdf]

Supplementary Table S1. TFs involved in regulation of TAA1/TARs-YUCCAs in other species

TFs	Genes	Species	Function	Ref.
EBB1	*YUC1*	*Prunus persica*	Bud break	[1]
TTG2	*YUC2*	*Brassica napus*	Salt stress	[2]
PIF4	*YUC2/5*	*Gossypium hirsutum*	Somatic embryogenesis	[3]
LBD16	*YUC2*	*Medicago truncatula*	Nodule organogenesis	[4]
CRF4a	*YUC4a/YUC4b/YUC10a*	*Glycine max*	Stem elongation	[5]
REV	*YUC5*	*Caragana korshinskii*	Drought stress	[6]
HEC1	*YUC4*	*Cucumis sativus*	Fruit neck length	[7]
HB1	*YUC5/TAA1*	*Prunus mume*	Apical meristem development and branching	[8]
ARR10	*YUC4/YUC5*	*Populus trichocarpa*	Drought stress	[9]

Abbreviations: EARLY BUD-BREAK 1, EBB1; TRANSPARENT TESTA GLABRA 2, TTG2; HYTOCHROME INTERACTING FACTOR 4, PIF4; LOB DOMAIN-CONTAINING PROTEIN 16, LBD16; CYTOKININ RESPONSE FACTOR 4a, CRF4a; HECATE 1, HEC1; ARABIDOPSIS RESPONSE REGULATOR 10, ARR10

References

1. Zhao, X.; Wen, B.; Li, C.; Liu, L.; Chen, X.; Li, D.; Li, L.; Fu, X. PpEBB1 directly binds to the GCC box-like element of auxin biosynthesis related genes. *Plant Sci* **2021,** 306, 110874.
2. Li, Q.; Yin, M.; Li, Y.; Fan, C.; Yang, Q.; Wu, J.; Zhang, C.; Wang, H.; Zhou, Y. Expression of *Brassica napus* TTG2, a regulator of trichome development, increases plant sensitivity to salt stress by suppressing the expression of auxin biosynthesis genes. *J. Exp. Bot.* **2015,** 66, (19), 5821-36.
3. Min, L.; Hu, Q.; Li, Y.; Xu, J.; Ma, Y.; Zhu, L.; Yang, X.; Zhang, X. LEAFY COTYLEDON1-CASEIN KINASE I-TCP15-PHYTOCHROME INTERACTING FACTOR4 network regulates somatic embryogenesis by regulating auxin homeostasis. *Plant Physiol* **2015,** 169, (4), 2805-21.
4. Schiessl, K.; Lilley, J. L. S.; Lee, T.; Tamvakis, I.; Kohlen, W.; Bailey, P. C.; Thomas, A.; Luptak, J.; Ramakrishnan, K.; Carpenter, M. D.; Mysore, K. S.; Wen, J.; Ahnert, S.; Grieneisen, V. A.; Oldroyd, G. E. D. NODULE INCEPTION recruits the lateral root developmental program for symbiotic nodule organogenesis in *Medicago truncatula*. *Curr Biol* **2019,** 29, (21), 3657-3668 e5.
5. Xu, Z.; Wang, R.; Kong, K.; Begum, N.; Almakas, A.; Liu, J.; Li, H.; Liu, B.; Zhao, T.; Zhao, T. An APETALA2/ethylene responsive factor transcription factor GmCRF4a regulates plant height and auxin biosynthesis in soybean. *Front Plant Sci* **2022,** 13, 983650.
6. Li, J. Y.; Ren, J. J.; Zhang, T. X.; Cui, J. H.; Gong, C. M. CkREV enhances the drought resistance of *Caragana korshinskii* through regulating the expression of auxin synthetase gene *CkYUC5*. *Int. J. Mol. Sci.* **2022,** 23, (11).
7. Wang, Z.; Zhou, Z.; Wang, L.; Yan, S.; Cheng, Z.; Liu, X.; Han, L.; Chen, G.; Wang, S.; Song, W.; Chen, J.; Liu, L.; Song, X.; Yan, L.; Zhao, J.; Zhang, X. The CsHEC1-CsOVATE module contributes to fruit neck length variation via modulating auxin biosynthesis in cucumber. *Proc Natl Acad Sci U S A* **2022,** 119, (39), e2209717119.
8. Li, L. L.; Zheng, T. C.; Li, P.; Liu, W. C.; Qiu, L. K.; Wang, J.; Cheng, T. R.; Zhang, Q. X. Integrative analysis of HD-Zip III gene PmHB1 contribute to the plant architecture in Prunus mume. *Sci Hortic-Amsterdam* **2022,** 293.

9. Wang, H. L.; Yang, Q.; Tan, S. Y.; Wang, T.; Zhang, Y.; Yang, Y. L.; Yin, W. L.; Xia, X. L.; Guo, H. W.; Li, Z. H. Regulation of cytokinin biosynthesis using *PtRD26*$_{pro}$*-IPT* module improves drought tolerance through PtARR10-PtYUC4/5-mediated reactive oxygen species removal in Populus. *J. Integr. Plant Biol.* **2022,** 64, (3), 771-786.
